# Tuberculosis infection among health care workers: a case series in two district hospitals, Kenya, August 2013

**DOI:** 10.11604/pamj.supp.2017.28.1.8222

**Published:** 2017-11-03

**Authors:** Evalyne Wambui Kanyina, Waqo Gufu Boru, Gerald Mburu Mucheru, Samuel Anyangu Amwayi, Tura Galgalo

**Affiliations:** 1 Kenya Field Epidemiology and Laboratory Training Program, Ministry of Health, Nairobi, Kenya; 2 East Africa Public Health Laboratory Networking Project, Ministry of Health, Nairobi, Kenya; 3 US Centers for Disease Control and Prevention, Kenya, Village Market, Nairobi, Kenya

**Keywords:** Infection prevention control, Health Care Workers, tuberculosis, Kenya

## Abstract

**Introduction:**

health care workers (HCWs) have an increased risk of M. tuberculosis infection and tuberculosis (TB) disease compared to the general population. We evaluated the magnitude of TB disease among HCWs in two District Hospitals in Kenya.

**Methods:**

retrospective review of TB laboratory registers was performed at Makindu and Kiambu district hospitals. Cases were HCWs with confirmed TB diagnosis working at either hospital from 2010 to 2013. Cases were interviewed using structured questionnaire to collect clinical and epidemiologic information. Infection prevention (IP) practices were observed and recorded.

**Results:**

Makindu and Kiambu had 91 and 450 HCWs respectively. As from the registers, 6,275 sputum smears were examined with 1,122 (18%) acid alcohol fast bacilli smear positive. Kiambu and Makindu reported 11 and five cases of TB among HCWs respectively. Of the 16, 57% were male; mean age was 45 (SD 5.32) years. HCWs affected were: four (25%) laboratory technicians, four (25%) nurses, two (13%) occupation therapists, two (13%) clinical officers and one pharmacist, telephone operator, driver and casual worker. Mean working time lost recuperating was 14 (range: 0-28) weeks. Both facilities lacked high-efficiency particulate air filters and Kiambu hospital lacked a biosafety cabinet too. Windows at both facilities were often closed and suspected TB patients shared common crowded outpatient waiting area where sputum was also collected. No standard reporting tool for TB disease among HCWs was in place at both facilities.

**Conclusion:**

TB disease was distributed across professional cadres with long working time lost recuperating. Inadequate IP measures exposed HCWs to occupational risk of acquiring TB disease.

## Introduction

Tuberculosis (TB) remains a major cause of morbidity and mortality globally. Kenya is one of 22 high TB burden countries [1]. Transmission risk of TB is influenced by factors such as the local TB prevalence, TB patient population characteristics, the setting, exposure risks such as occupation, and effectiveness of TB infection control measures [2]. In a hospital setting, the risk of transmission from individuals infected with TB to other patients and to health care workers (HCWs) is well recognized [3, 4]. Specific occupation cadres including medical doctors, nurses, clinical officers, paramedics, radiology technicians, patients and wards attendants have a higher risk of TB disease [5]. Infection control practices, such as prompt diagnosis and treatment of infectious cases, effective ventilation systems, isolation rooms, cough corners, access to and use of personal protective equipment (PPE) and surveillance, are also factors in the spread of TB [3,6,7].

In August 2013, reports of an increase in cases of TB among laboratory technicians from one to three since 2011 to 2012 were received at the Kenyan chief laboratory technologist’s office. This prompted the Ministry of Health to conduct an investigation at two district hospitals, Makindu and Kiambu Hospitals, to confirm and investigate the magnitude of TB disease among HCWs, and to document lapses in infection prevention practices.

## Methods

We conducted this investigation between 19th and 22nd August 2013. A case of active TB disease among HCWs was defined as any HCW working at either Makindu or Kiambu Hospital during 2010 to 2013 with confirmed TB diagnosis either by sputum microscopy, sputum geneXpert analysis, sputum culture that isolated *m. tuberculosis* or chest radiography. Available TB cases were interviewed using a structured questionnaire focused on clinical and epidemiologic information, as well as occupational exposure. In both hospitals we performed a retrospective record review of acid alcohol fast bacilli (AAFB) laboratory registers from 2012 to 2013 and we reviewed chest clinic records from 2010 to 2013. Infection prevention practices employed in the two hospitals were observed and recorded. Data were analyzed using Epi info version 3.5.1 software.

As a public health response by the Kenyan Ministry of Health, no review by an institutional ethics review board was sought. However, the protocol was approved by the Kenyan Field Epidemiology and Laboratory Training Program, Ministry of Health and both hospitals. All study participants gave an informed consent before participation. Participants were assign a participant identification number (PIN). This ensured anonymity for the study participants. Each questionnaire was labeled with the participant’s PIN and not personal identifiers. Access to the computer used for storage of study data was limited to the investigators only. The computer and study data was protected by means of a password.

## Results

**Figure 1:**
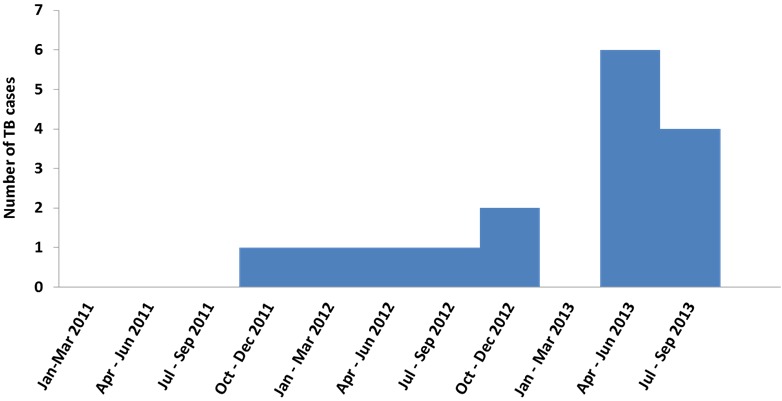
number of human and veterinary health units that had laboratory facilities or services, and the number of units visitedes Studies

**Figure 2:**
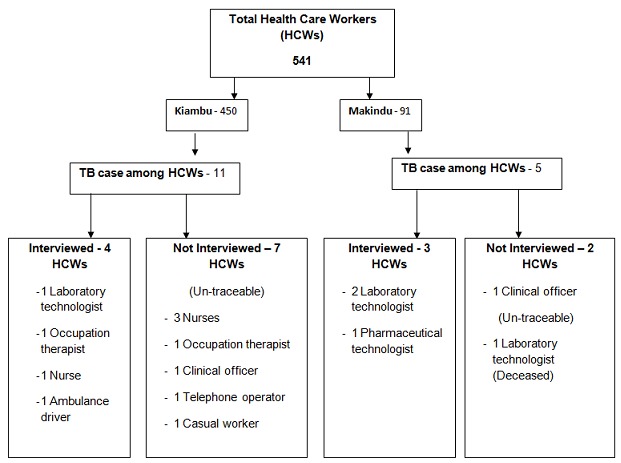
epicurve of TB cases among health care workers, Kiambu and Makindu District Hospital, Kenya, 2010-2013

We identified 16 cases of TB disease in total, with five TB cases among 91 HCWs (5.5%) in Makindu Hospital and 11 TB cases among 450 HCWs (2.4%) in Kiambu Hospital (Figure 1). Nine (57%) TB cases were male and seven (43%) TB cases were female, with a mean age of 45 years (SD: 5.32) among all TB cases. The 16 TB cases were distributed across all HCWs cadres; four (25%) laboratory technicians, four (25%) nurses, two (13%) occupation therapists, two (13%) clinical officers and one pharmacist, telephone operator, driver and casual laborer (Figure 2). Cases were working in different hospital departments; five (31%) in TB clinic, four (25%) in microbiology department, four (25%) in wards, two (13%) in administration and one (6%) served the entire hospital. Based on the total numbers of HCWs in each cadre in both hospitals, the proportion of TB cases was higher among the laboratory officers (16%, 4 of 25) and occupation therapy officers (15%, 2 of 13) and lower among clinical officers (7%, 2 of 14) and nurses (1.4%, 4 of 285). One death was reported in a case that had TB-Diabetes co-morbidity. Autopsy report was not availed. This was the only co-infection reported.

Nine TB cases were not available for interview; three were not on duty, five had been transferred to other health facilities and one was deceased. Among seven cases interviewed, five (71%) had been diagnosed using radiology and two (29%) by smear microscopy technique. The mean hospitalization duration was 16 days. Weeks of service reportedly lost while recuperating from TB disease ranged from two to 28 weeks (mean 14 weeks).

All the laboratory technicians that were diagnosed as TB cases during 2010–2013 had worked on the laboratory’s AAFB smear microscopy bench. Both of the occupation therapists that became TB cases reported attending to TB patients at the time of their diagnosis. None of the cases interviewed had been trained on infection prevention control (IPC) practices in the previous one year. None of the TB cases reported any known active TB disease cases among their close contacts.

### TB diagnosis workload

**Figure 3:**
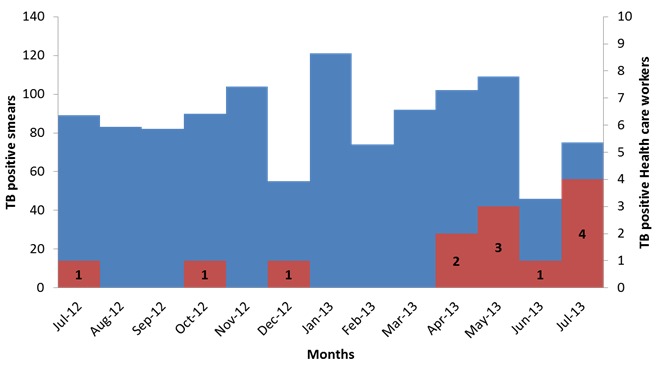
epicurve of TB positive smears and TB cases among the Health care workers, Kiambu and Makindu District Hospital, Kenya, 2010-2013

During the period for which record were reviewed, 6,275 sputum smears had been examined and 1,122 (18%) of these were positive (Figure 3). There were 885 (79%) positive smears from 4,338 smears in Kiambu Hospital and 237 (21%) positive from 1,937 smears (31%) from Makindu Hospital. In Kiambu, there were an average of 362 smears per month; 74 (20%) positive smears per month and approximately 4 smears positive per day. 1959 new cases were examined in Kiambu, 20% (384) were positive for TB. On the other hand, Makindu had an average of 162 smears per month; 20 positive smears per month and approximately 1 positive smear per day. 988 new cases were examined for TB in Makindu of which 12% (114) were positive for TB.

### Infection prevention control practices

No standard reporting tool for TB disease among HCW was in place at the time of the investigation. All HCWs were using coats and gloves when handling patients, specimens and contaminated materials; however, there were no high-efficiency particulate air filters (HEPA) masks being used at either facility. Although surgical masks, as part of standard personal protective equipment (PPE) at both hospitals, were available, we observed that some HCWs from both in-patient and-outpatient departments did not use them when treating TB patients. We observed poor adherence to hand hygiene recommended guidelines [8]. The necessary infrastructure for optimal hand hygiene such as dedicated hand washing sinks, liquid soap and single use disposable paper towels for drying hands were not available for patients and/or HCWs in Kiambu Hospital. Dedicated hand washing sinks and liquid soap were available in the administration and laboratory department at Makindu Hospital.

Kiambu Hospital’s laboratory sputum testing area lacked a biosafety cabinet. Both hospitals did not perform an infection prevention performance audit and efficacy test for disinfectants used. Neither laboratories had a biosafety officer, biosafety manual, emergency response/contingency plan and occurrence book. Windows at both facilities were often closed both in the in-and-outpatient departments, which limited ventilation of the rooms. At both hospitals, suspected TB patients shared a common crowded outpatient waiting area with other patients where sputum specimens were also collected. There was no consistent TB-triage by nurses during patient admission and suspected TB cases were not included in TB-IPC measures such as isolation and use of PPE in both hospitals.

## Discussion

Our investigation identified cases of TB disease in both hospitals distributed among all HCWs cadres. This is in agreement with Tura et al. findings that any hospital staff that spend time in a room with a patient is at risk of TB infection irrespective of their designation [9]. However, most HCWs TB cases are reported among those directly involved in patient care [10]. Our study reported lower rate of TB cases in nurses compared to other cadres. Kiambu Hospital had a higher TB workload and TB smear positivity but lower TB cases among HCWs compared to Makindu Hospital. At both hospitals, effective TB-IPC practices by HCWs were hampered by the lack of TB-IPC training and appropriate infrastructure.

In our study, all the laboratory and occupation therapy officers were diagnosed with TB while working with suspected TB patients. TB was reported to be the sixth most common occupationally acquired infection among laboratory workers [11] and it has been estimated that laboratory workers have a two to nine times higher risk of contracting TB than the general public [12]. This is mainly due to tubercle bacilli aerosolization during testing procedures [6]. Similar to our findings, Cuhadaroglu et al. reported a lower incidence of TB among nurses compared to clinicians such as clinical officers and doctors in Turkey [13]. This might be because nurses in Kenya work in mainly in departments where the TB status of patients is known and to whom they are providing treatment, and they can therefore take steps to protect themselves against infection. On the contrary, other cadres in Kenya (laboratory, occupation therapy officers, clinical officers and doctors) more commonly work in departments where patient TB status is undisclosed or undiagnosed, leading to increased risk of infection.

Nosocomial TB transmission among HCWs remains a strong risk factor for exposure to TB infection [5]. Prevention and management of TB infection is the responsibility of all health care staff working and an integral element of patient and HCWs safety programmes. Areas where TB patient care and diagnosis were provided, including TB clinics, wards and microbiology laboratory departments, reported high rates of staff with TB disease. Both of the hospitals in this study had shortcomings in patient and HCWs safety schemes. Cough corners and isolation areas for TB patients were not available in either facility. Hand washing hygiene and ventilation guidelines in infection prevention were not observed, which are known to increase the risk of nosocomial infection. Adherence and consistency in use of infection prevention strategies have been shown to minimize healthcare associated infection risks by 27% to 81% based on the country’s TB incidence [14] and reduce the frequency of pseudo-outbreaks [15]. According to internationally agreed standards, IPC practices should include: rapid clinical evaluation (TB-triage) of all persons (staff, patients and visitors) with symptoms suggestive of TB, segregation of patients with known or suspected pulmonary TB, use of effective local exhaust ventilation in connection with high-risk procedures, staff training, and ongoing risk assessment [16]. These measures emphasize the need for the implementation and adherence to infection control strategies which could reduce the number of staff and other patients who come into contact with aerosolized *Mycobacterium tuberculosis.*

Our study has several limitations. First, although HCWs did not recall or report any known close contacts with TB, we cannot rule out that TB exposure did not occurring outside of the hospital by exposure. We cannot also rule out that HCWs could infect each other outside of the work setting, if they are symptomatic, and if they also have social contacts with each other. Genetic sequencing of *M tuberculosis* isolates could help differentiate nosocomial versus community spread of TB, however this technology is not commonly done yet in high prevalence TB settings and isolates are not typically stored for later comparison. The two hospitals were conveniently selected, rather than randomly chosen. Information on the HIV status of the cases was not volunteered. This theoretically limits representativeness and generalizability. Only a small number of HCWs with confirmed TB disease were available for interview.

## Conclusion

TB disease was found among all professional cadres in the two hospitals investigated. There was an increase in TB cases among heath care workers in the second (April – June) and third (July – September) quarter of 2013 compared to 2012. HCWs in clinical departments were more affected than those in the administrative department. Inadequate infection prevention measures, lack of TB-IPC training and poor laboratory quality management system, likely exposed HCWs to occupational risk of acquiring TB disease. The lack of basic measures for airborne IPC, combined with the high TB positivity among patients, leads us to suspect there was nosocomial transmission of TB among HCWs in these hospitals. We recommended the enforcement of infection prevention control measures implementation such as ensuring the supply of HEPA mask and improve ventilation systems in the health facilities, biosafety/infection control/TB trainings and strengthening the laboratory quality management system by putting in place biosafety manual, standard operating procedure, contingency plan, incident/occurrence forms/records and calibration/maintenance/validation reports.

### What is known about this topic

Health care workers (HCWs) faces a higher risk of acquiring tuberculosis (TB) compared to the general population;

The increased risk of TB among HCWs is attributed to close contact with TB patients and TB specimens in the line of duty;

Implementation and adherence to infection prevention control measures in hospital settings reduces occupational-associated TB infections.

### What this study adds

This study documents that HCWs of all designations faces the risk of acquiring TB disease;

It outlines the need to enforce implementation of infection control strategies in the public hospitals in Kenya;

It also highlights the need to put in place a standard reporting tool for TB disease among HCW.

## Competing interests

The authors declare no competing interest.
